# "The Hospital" Nursing Mirror

**Published:** 1896-12-26

**Authors:** 


### "The Hospital" Nursing Mirror.

Being the Nursing Section of "The Hospital."

[Contributions for this Section of "The Hospital" should be addressed to the Editor, The Hospital, 28 & 29, Southampton Street, Strand, London, W.C., and should have the word "Nursing" plainly written in left-hand top corner of the envelope.]

### News from the Nursing World.

### THE QUEEN'S COMMEMORATION.

At Grosvenor House, on December 16th, by invitation of the Duke of Westminster, a preliminary meeting was held in support of the scheme to celebrate the sixtieth year of the Queen's reign by an effort on behalf of the Queen Victoria Jubilee Institute for Nurses. The meeting was a representative one, and well attended. The Archbishop-Elect of Canterbury was present, the Chief Rabbi, Cardinal Vaughan, the Earls of Strafford and Lauderdale, and the lord lieutenants and mayors of various counties and towns. A letter was read from Miss Nightingale, warmly approving of the scheme, and promising her assistance. The Hon. Sydney Holland, who is hon. treasurer of the fund, announced that, although subscriptions had not yet been asked for, no less than £18,000 had already been promised. A resolution approving of the creation of local committees to aid the fund was carried, and some interesting speeches made. The meeting concluded with the election of the Executive Committee.

### WORKHOUSE NURSING.

Mrs. Roundell's paper on "Nursing in Workhouses," read by Mr. C. Roundell at the South-Eastern Poor-law Conference, well summed up the impossibilities of the present system. It is recognised by all who know anything of the matter that "the sum and substance of the workhouse nursing difficulty" is the relation of the trained nurse to the untrained matron, and the continuance of the anomalous arrangement by which the wife of the master necessarily holds the latter position, the master and matron being, in the eyes of the Local Government Board, "the unit upon which the whole workhouse administration turned." Mrs. Roundell has studied the matter in all its bearings, and her remarks thereon may well be recommended to the consideration of the Local Government Board.

### LEEDS NURSING ASSOCIATION.

The Leeds District Nursing Association has just received a very pleasant testimony to the value of its work among the poor. The *Yorkshire Daily Post* says that "a lady in a humble position in life," seeing the help and comfort brought by the nurses in their daily visits to her sick neighbours, determined some years ago that if it should ever be in her power she would help the association. Having lately received a legacy she at once sent a donation of £50 to Miss Garlick, the honorary secretary of the association.

### CHRISTMAS AT THE CHILDREN'S HOSPITAL, DUBLIN.

The little patients at the Children's Hospital, Temple Street, Dublin, are looking forward to "a good time" this week, for "Father Christmas" is expected, and he will not leave the building till the occupant of every cot has received a present of some kind. Then there is to be a tree in each ward, and the dressing of these trees and the distribution of the gifts delight the children's hearts. A great effort is being made to clear off a debt on the Children's Hospital, and to help this a grand *fête* is to be held next spring in the Rotunda Rooms and Garden.

### SECTARIANISM.

It has been so much more the custom of late to do away with all sectarian distinctions in institutions for the care of the sick that it is disappointing to find the old "religious disability" question cropping up again in connexion with infirmary nursing. The Cheltenham Board of Guardians have lately declined to appoint a nurse recommended to them by the Workhouse Infirmary Nursing Association on the ground of her being a Roman Catholic, and more recently a similar objection was raised by two lady guardians of St. Austell. In the latter instance, however, the common-sense of the Board fortunately prevailed, and the proposal to rescind the appointment of the nurse was withdrawn.

### STUDENTS IN THE WARDS.

At the annual meeting of the Westbourne Provident Dispensary, Mr. Edmund Owen, surgeon to St. Mary's Hospital, spoke of the objections sometimes raised to the presence of students in hospital wards. This, he said, was founded on a great mistake. No hospital was complete without students, and their presence was of the greatest advantage to the patients, for their presence made the staff take unceasing pains to understand the patients' diseases. It was impossible for a surgeon, for instance, to teach a class of students unless he fully understood the patient's case. He thought that if patients recognised this they would never object to students at the bedside, but would rather feel that their presence was a guarantee that their case would be thoroughly worked out and judiciously dealt with. We quite endorse this view of the matter, and we would carry it further and say that the presence of students of another kind is also very advantageous to patients in hospitals. Nurse probationers are students of nursing, and their presence has the same influence on the character of the nursing as that of medical students has on the character of the medical work. Students, whether of medicine or nursing, are extremely critical. Rough experience has not dulled their enthusiasm or lowered their ideals, and their presence in the wards keeps up an all round standard both in medicine and in nursing which might be difficult to maintain were the work not done under the eye of such an exacting form of public opinion.

### CHRISTMAS IN THE HOSPITALS.

There are plenty of signs that Christmas festivities have already begun with vigour in the metropolitan hospitals, and invitations to teas and trees, concerts and carols, are coming in gaily. Unfortunately many of these pleasant little entertainments fall on the same day and hour, so we must rely on our readers sending us in any notices they wish to see in The Hospital as promptly as possible, and intimations of forthcoming entertainments, too, should be despatched without delay, or they may be too late for insertion.

### CONCERT AT CHELSEA INFIRMARY.

A VERY pleasant concert was got up recently for the patients in the Chelsea Infirmary by Miss Mary Willoughby, assisted by a number of friends. The nurses' dining hall makes an excellent place for such an entertainment, and the enjoyment of the audience was keen throughout the evening. Miss Willoughby is intending to provide a series of these entertainments through the winter, and if they are all as successful as this one, she will have contrived to give a great deal of pleasure.

### OUR CHRISTMAS GIFTS.

SOME delightful parcels of clothing reached THE HOSPITAL office last week, for which we have to thank the senders very gratefully. We have also received several contributions in money, and as no prizes have been given this year we are spending a certain sum in the purchase of warm vests and shirts, and other things that will be helpful to poor patients leaving hospital without proper clothing. Our kind contributors would feel well repaid for the trouble they have spent in fashioning petticoats, jackets, andshirts, and knitting socks and stockings, could they follow their handiwork to the hospitals and see with what satisfaction the garments are there received,

### EVELINA HOSPITAL FOR CHILDREN.

THE Evelina Hospital for Sick Children, which has been closed for three months for refurnishing and decorating, is just ready again for the reception of patients, and invitations to a general inspection were issued for Wednesday in last week. The wards, out-patients' department, and theatre have been painted, and the walls lined with white tiles to a height of five feet from the ground; new furniture has been added where needed, and the whole building fitted with electric light. A new wing is about to be erected on an adjoining piece of land presented to the hospital by its president, Baron Ferdinand de Rothschild, who has also given a donation of £10,000 towards the expenses of the recent improvements and the cost of the further enlargement. When this addition is completed the number of available beds will be increased to 100.

### SHORT ITEMS.

THE Queen has conferred the Royal Red Cross upon Sister Sarah Elizabeth Oram, of the Army Nursing Service, in recognition of her work among the sick and wounded sent to Cairo during the late fighting in the Soudan.—An anonymous donor has sent £500 to Queen Charlotte's Lying-in Hospital, Marylebone Road, in aid of the Extension and Improvement Fund.—Miss Longmore, the retiring matron of the Monkwearmouth and Southwick Hospital, was presented on her departure with a pretty inlaid Chippendale writing table from a number of friends in Sunderland and Monkwearmouth with expressions of great regret "at the loss of one who had won their esteem and admiration."—Mrs. Marshall's School of Cookery held an exhibition at the Queen's Hall on December 17th and 18th. A number of prizes and medals were offered for competition, amongst others several by Liebig's Extract of Meat Company for the three best dishes containing their preparations.—Last week the Venetian Part Singers paid a visit to Westminster Hospital and gave the patients much pleasure by singing a number of glees and part songs in the wards.—The Ladies' Kennel Association, through the Countess of Ilchester, has sent £150 to the West-end Hospital for Paralysis and Epilepsy and £50 to the Home for Crippled Boys, Kensington, being the profits from the Dog Show held by the association at Holland House in the summer.

### Christmas in the East End.

MANY inhabitants of the West End of London contrive to spend at least part of the Christmas holidays away from its leaden skies, mud, and fog. In fact the wealthier classes who live, nominally, *in* London, manage to spend a considerable portion of the year *out* of it.

But the odd million who exist in the East End may be found at home throughout the season of Christmas, as well as at most other times of the year. Strange as it may seem to those who look with inexperienced eyes at the wretchedness and poverty of this part of London, the festival is anticipated and—in a certain way—enjoyed there. The tawdry, cheap decorations which herald Christmas, often weeks in advance, in the poorest of East End shop windows, in advance, in the poorest of East End shop windows, are a joy to beholders. The frail festoons of coloured paper, the unnatural imitation-holly, the coloured tapers, and the crackers make a centre of attraction in even the shabbiest of bye-ways.

The outward and visible signs of something, which is often hardly more than a name to them, become perhaps confused with that intangible "something" itself. For instance, the East-ender, catching sight of a spray of real holly, says, "That's the first bit of Christmas as I've seen," and it means actual Christmas to him. The lonely fishermen in the North Sea use the same expression, and will go for miles, often in peril of life and limb, to get a "bit o' Christmas," *i.e.*, spray of holly from a hospital mission ship.

It may be safely asserted that to many a true East-ender the signs of Christmas-tide are the only part of the festival of which they have knowledge. Hence, those who study the phases of life laid bare to observant eyes in every lane and court, can see parents and children alike keenly awake to every sign of the coming festival.

Never a crate of "greenery" is delivered at institution, church, or private house without some ragged urchin or draggle-tailed lassie collecting the spoil of scattered berries or broken sprays. The workman will stoop his weary shoulders to pick up the bough fallen from an overloaded cart; or else he expends his hardly-earned coppers in a "bit o' Christmas" from a street stall.

The careworn mother, with her scanty purchases held closely under her shabby shawl, can be seen standing irresolute before the tempting display on the truck. Those meagre bundles of green are not cheap, and certainly their quality is not first class, but they seem idealised for the moment and, if there be a stray coin in the poor woman's thin hand, it will be used to purchase a single "lot" which she will select for herself after a prolonged and critical inspection of the whole stock. In some of the "homes" at the East End (and what a farce it seems to use the beautiful word in this connection !) Christmas brings no alleviation of the daily burden. There are, however, but few to which a vague "keeping" of the festival fails to penetrate.

Children are bidden to great feasts provided by their wealthier brethren ; and Susie's "tea party at the 'all," or Tom's dinner at some other public institution, shed a reflected glory on the family at home.

Doles of food, or clothes, or money are all possibilities within the ken of the poorest of the poor, and the least scrupulous have ways and means of becoming unsuspected partakers of various benefits to which they have no just claim. The decent poor make a brave struggle to save up for Christmas, to obtain, in fact, a little extra store which may provide a bare sufficiency for so-called "Christmas fare."

The sick put off going into hospital until after Christmas if they can, and many ask the doctor's permission to leave before the Eve. In cases where acute disease or accident makes immediate admission to the wards compulsory, most patients find their self-pity turned into congratulation. Piles of evergreens beyond their wildest dreams; gifts of all kinds; such special fare as may be sanctioned by the doctor, and, over all, a spirit of goodwill which makes the trivial acts of kindness doubly welcome.

In the years to come those weary, careworn, East-enders speak as cheerily as their own youngsters of that eventful season "when I was laid up in the 'orspital and 'ad a rare good time."

### Royal British Nurses' Association.

### ANNUAL CONVERSAZIONE.

THE Executive of the Royal British Nurses' Association are to be congratulated on their choice of the Portman Rooms frr the annual conversazione held on Friday, the 18th, where the ample space lent itself well to a large gathering of guests, members of the association, and friends—some 1,500 in all. Sir James Crichton Browne, Chairman of the Executive Committee, Mr. Langton, Mr. Fardon, Miss Thorold, and Mrs. Dacre Craven received the guests in the first room, the upper end of which was arranged for the concert which was to succeed the distribution of badges. H.R.H. Princess Christian, looking very well, and handsomely dressed in petunia velvet, appeared on the platform soon after nine o'clock, and was presented by Miss Myra Langton with a beautiful bouquet. Besides the members of the Executive Committee and the honorary officers, there were present amongst the guests: Lord and Lady Halsbury, Sir Francis and Lady Jeune, Sir William Broadbent, Sir Henry Thompson, and Miss Peter, Inspector of the Q.V.J.I. Mr. Clinton Dent made a brief speech by way of introduction, and the Princess then presented their badges to the newly-enrolled members of the association, who, by-the-way, would have been the better for a little previous drilling. Afterwards an enjoyable concert was given, in which the Border Minstrels Ladies' Orchestra took part, and to the success of which M. Johannes Wolff, Mdlle. Giulia Ravogli, Miss Helen Jackson, Mr. H. Wharton Wells, and others contributed. It was a pity that a little want of management led to quite unnecessary crowding in the doorways leading through to the tea-rooms, so that not even the distant strains of M. Wolff's violin or the knowledge that Ravogli was singing could lead those who had already reached the supper stage to face the scramble back to the concert-room. A sprinkling of nurses' uniforms always has a pretty effect, and there were many varieties to be seen on Friday, from the regulation dark blue to the more attractive pinks and light colours of private nurses and probationers. Looking at the nurses present with a critical eye, there was certainly an improvement in "heads" this year, and the disappearance of the "bun" is a thing to be rejoiced over. Would that the fringe might go the same way! Why, too, cannot nurses keep off "shop" when at a social gathering? A lay visitor on Friday night was entertained by some ward reminiscences from two nurses just beside her, which must have considerably interfered with a due subsequent enjoyment of the very excellent refreshments always provided on these occasions.

### Appointments.

ULVERSTON AND DISTRICT COTTAGE HOSPITAL.—Miss Ethel Gray has been appointed to the post of Matron at this hospital. Miss Gray received her training at the Royal Infirmary, Edinburgh, and afterwards worked at the Longmoor Hospital, Edinburgh. She has also had experience in private nursing.

HUNTINGDON COUNTY HOSPITAL.—Miss Beatrice Carr has been appointed Matron at the Huntingdon County Hospital. She trained for three years at the Sunderland Infirmary, afterwards qualifying as a Queen's Nurse, and working at Peterborough both as district nurse and taking temporary charge of the infirmary there. Miss Carr joined H.R.H. Princess Christian's Trained Nurses' Home at Windsor, and latterly has been doing district work at Huntingdon and Godmanchester.

### Christmas in a Country Workhouse Infirmary.

By A NURSE.

CHRISTMAS festivities penetrate in a mild degree even to the quiet interior of a country workhouse infirmary, and the very fact that so many of our patients are without relations or friends makes us, as nurses, feel we must do all we can to make up for that want, and cheer our poor folks as much as possible at this season. Preparations begin weeks beforehand; mottoes and texts are made under the master's directions ready for the great occasion, and people around are asked to send evergreens in good time for the decorations. On Christmas Eve some of our ever-kind lady guardians, who are unable to put in an appearance on Christmas Day itself, arrive with their gifts, which are left with the matron. The work is all completed, the wards made bright and pretty, and each one is the richer for some special gift brought by the ladies. These are one and all most acceptable; nice plants in pots, well-framed pictures, and really lovely table covers, so that the long ward tables look charming. A kind friend brought one year a number of dainty nightcaps and quantities of shawls, cuffs, and bed-socks, hand-worked, while yet another sends cards and letters for each patient. We had a delightful present once, in the shape of a dozen prettily-upholstered wicker chairs, and several people interested in the infirmary supply us with oranges, tea, sugar, tobacco, and other good things. On Christmas Day visitors come early, and many of the guardians help to serve the dinners of roast beef and pork, vegetables, pudding, and beer. Last year a party of members of the G.F.S. came and sang carols, accompanied by one of the nurses on the harmonium—a treat much appreciated, especially by the old men. Altogether, the day is a very happy one for all, and the nurses and officers feel well repaid for all their efforts in seeing the pleasure of their patients.

On New Year's Day we have always a great treat for the whole house—a Christmas tree and a tea, cakes of the very nicest, and bread and butter of the daintiest, unlimited jam, and other nice things. This year we are to have a concert also, so our poor people will have a pleasant and cheerful time if all goes well.

### Novelties for Nurses.

### NOVELTIES AT MESSRS. DEBENHAM AND FREEBODY'S.

We do not believe that any establishment has in so short a space of time achieved for itself such popularity in the nursing world as Messrs. Debenham and Freebody, Wigmore Street. The department which they have opened for nurses' uniforms is of comparatively recent date, but owing to the excellent manner in which it is conducted has won the confidence and approval of those whose custom it has succeeded in obtaining. We have received samples of their materials for nurses' dresses, and have no hesitation in pronouncing them to be the best and cheapest for the money we have had the pleasure of seeing. The Oxfords at 6½d. are wonderful. The colours are good and warranted fast. There is among them a lovely rose colour, and a soft blue that is certain to be a favourite. The striped regattas at the same price are likewise to be recommended, also the galateas, which have some excellent designs among them. Irish coloured linens run into a little more money, but are well worth it from their superior finish and the general excellence of the manufacture. Drills and prints are also very tempting; of the latter there are some charming patterns, in narrow striped blue, pink, mauve, and lavender. The shades and qualities of the various zephyrs are numerous, the colours are beautiful and the prices most reasonable. Our readers will be well advised to send at once for patterns or pay a visit to the department, where nurses are always welcome.

### "Admitted as Urgent."

THE great strike of 18— was specially felt in our district. It was followed by severe frost, and with Christmas came famine. Hunger-stricken patients filled our wards and overflowed into odd corners. At last the fiat went forth that no more medical cases were to be admitted. Things were even worse at the workhouse infirmary, which an evening paper in a sensational article described as "A Modern Black Hole of Calcutta."

Every day at noon food was distributed to the starving poor from a number of vans drawn up opposite the municipal offices. A couple of days before Christmas I formed one of the crowd of spectators lining three sides of the public square. Squads of haggard men filled the greater part of the open space and tailed away into the side streets. The women were marshalled separately near a mission van specially reserved for them. On the previous day the supply of food had proved inadequate, only the first comers being served. Hence, when the town-hall clock struck twelve, the men suddenly broke rank and made a rush for the vans. A painful scene followed. The body of the square was filled with a struggling mass of ravenous men, each bent on obtaining food at any cost. Some of the distributors pitched loaves among the crowd to save their vans from being looted, and wherever a loaf fell human beings fought like wolves for the fragments. Standing beside me, intently watching the conflict, were a boy and girl whose scanty rags barely covered their shivering bodies.

The boy, aged, perhaps, fourteen, carried a workman's tea-can. His companion might have been a year older. She was thick set—almost stunted—with a broad, sensible face, already marked by care.

"It ain't no use, Jim," I heard her say to her companion, "talkin' about the woman's van. He don't like its soup. It's from the red one you must get it." I looked towards the red van. Round it the human tide surged most fiercely. I would have stopped the boy, but he was already worming his way into the crowd. It seemed almost certain that he would be crushed to death, but in about ten minutes he re-appeared and hurried towards the girl. "I got it, Mary!" he called out exultingly, "and saved more'n half." "Yes," she said, taking the can and sniffing the steam. "It's the proper stuff. If Mat don't drink it all I'll keep the rest for you. Now you go to the woman's van for some bread, an' I'll go back at once with the soup to Mat." Jim had probably little difficulty in obtaining the bread, for the women stood aside on that day of trial that the children might be served first. Meanwhile the girl Mary, having wrapped up the can in her cotton neckerchief, was trudging sturdily in the direction of the hospital with her head bent to the biting wind. I followed, and was about to speak to her as we were crossing a patch of waste land, when, suddenly leaving the path, she entered an open wooden shed which had been built against the side-wall of a disused workshop. In the shed was an old covered-in open-fronted parcel van. As it was only a few yards from where I stood, I crossed to the shed and looked into the van. The girl was kneeling beside a child who sat among straw at the far end. Some movement on my part startled her, and the can of soup dropped from her hand, the contents being lost. She snatched it up, and after peering into it, cried despairingly, "Oh, Mat, what'll I do: it' all lost!" Then she suddenly turned, and crawling to the front of the van, faced me with blazing eyes. "It's all your fault!" she said angrily, "Poking your nose where you're not wanted." "I'm sorry" I said conciliatingly, "I didn't mean any harm. Perhaps I may be able to help you in some way." "We don't want no help. We're all right," she retorted. "I don't know about that," I replied. "From the look of that boy I believe he is very ill." "Do you?" she said rudely, "I know you. You're a nuss from the 'orspital, that's what you are. He aint got nothing but a cold, and isn't goin' to no 'orspital, so I tells you straight." During this parley her companion had come forward and was shyly watching me over her shoulder. He was a wisp of a child, some seven years of age, and the flush showing through the grime of his cheeks, his bright eyes and rapid breathing told a plain enough tale. "Poor little mite," I said. "He's right enough," insisted the girl, "And now you'd best go back to the 'orspital. He's goin' to stay with me, an' that's the last word." But she had the weakness of her sex, and as I stood in doubt, burst out again, "I know all about 'orspitals, I do. Manys a time before mother died I heard father a-tellin' her all the dreadful things they'd do to her if she went into one." "So you have a father?" I interjected, hoping to stem her flow of invective. "Yes," she answered, more soberly, "but last week we was turned out of Ten Men Court, an' father left us at the station an' never came back. Jim says he just wanted to lose us. Then we came here." It struck me that the van, with its lining of clean straw and its blanket of sacking neatly rolled up in one corner, was not a bad exchange for a cellar-room in Ten Men Court. Yet it was impossible to leave the boy in such a place. He was evidently in great need of medical treatment. "Sister will know," was my conclusion. We all went to Sister for advice and sympathy. A few minutes later I was in her room telling my story. She informed the house surgeon, and in due time I was summoned for cross-examination. "It is more than awkward," he said, after a pause. "There was a little—er—trouble about the last emergency case I admitted, and the infirmary is out of the question. However, it's only a step from here, so I'll just go and have a look at the little chap." As he disappeared, Sister smiled, and I read that she considered the case as good as admitted. I thought of Mary and doubted. Half-an-hour later the house surgeon came to our ward. "Sister," he said casually, "What about that broken cot that was removed to the lower regions?" "I can have it up in five minutes," she answered. "All right," he said, "Sam can go for him with a blanket." "And the girl?" Sister ventured. "She's a trump," he replied gravely. "She harangued me in fine style whenever I appeared on the scene. I was forced to tell her that if he remained in the van her brother's death would lie at her door, and that she was thinking more of herself than him. 'Very well,' she said, turning white; 'you ask him, and if he says "Yes" he can go.' Then she put her arms round him and waited. He cried at first, but began to pluck up when I told him about the toys he would have, and finally capitulated to the Christmas tree. When he said, 'I'll come,' the girl flung herself face downward in the straw. Then I thought it best to leave them."

"He 'pears clean an' comfortable," said Mary, with matronly gravity, as she stood by her brother's bed on Christmas morning. Then she looked down the brightly decorated ward, with its double row of cots, each with a happy little occupant, and her eyes filled with tears. "Nurse," she said, drawing a thin hand across her face, "I'm sorry for what I said about 'orspitals. Father must ha' been wrong."

J. B. AND M. D.

### Christmas Books.

BOOKS for school-boys! Their name is legion, if we may judge by the many samples before us now. The first to catch the eye is a finely got-up new edition of "Monte Christo,"* that book, the reading of whose wonder pages marks an epoch in the life of a boy, be he young or old. This edition is profusely illustrated, and its price makes it within the means of any purchaser.

In "Cassell's Family Magazine" for 1896† there is so much good reading as almost to disarm discussion, and although compiled especially for the general reader, there is much which seems directed to the intelligent boy reader, and would make it an acceptable present for Christmas. For the boy who would be a sailor (and what boy on the under side of twelve would not?) we commend "Every Inch a Sailor."‡ Scenes of the most daring description are to be found in these pages and illustrations (which may almost be classified as nightmares, by those persons who are so wanting in enthusiasm as not to be "moved" by the mighty spirit of the sea) accompany pages devoted to hairbreadth escapes. "Far Away in Arctic Seas" and "In the Wilds of Kamschatka," &c. So sensational is this book that no true-hearted sea-going boy will put it down without being further stimulated in his longing for adventures on the briny.

"To Central Africa on an Iceberg,"§ despite the fact that the hero of the adventurous romance is a white bear, is scarcely second in point of thrilling detail to the above story, but it has this further advantage that it is an original theme, treated in an original manner.

In "Three Girls in a Flat,"‖ we find Miss Heddle fulfilling a long-felt want as a writer for girls. Boys are well catered for, but a curious dearth of books suitable for young girl readers has been observable among publishers' lists. The story of these three girls, their struggles, endurances, independence, each in turn appeals to one's sympathy, and all alike are told in a bright and entertaining manner.

"A Humble Enterprise,"¶ by Ada Cambridge, though apparently aimed at the same class of readers as the above, fails in its endeavour, because it is neither calculated to entertain or to instruct. "The Little Larrikin" ** strikes us as one of the best books which have appeared this season. It is quite delightfully written, and has a distinctive character of its own. Miss Ethel Turner's book will, we predict, attain the popularity it richly deserves. For those readers who delight in mystery we commend "Iras,"†† by Theo. Douglas, which concerns the resuscitation to life of an ancient Egyptian mummy. The plot has an originality about it certainly. Talking of "plots," it is interesting to find a writer who has the hardihood to compose a novel "without a plot," but Curtis Yorke's "Because of the Child"‡‡ avowedly boasts of the omission. The tale forms a fourth volume of "The Daffodil Library," a series of novels which Jarrold and Sons are bringing out in dainty covers.

Mrs. Jebb, in her collection of stories about "Some Unconventional People,"§§ shows herself not only a keen observer, but capable, as few of her sex are capable, in bringing home a particular situation to her readers by a single touch. This gift, at once artistic and literary, distinguishes in a rare degree the various descriptions in "Some Unconventional People." Cassell and Co. are the publishers of Mr. J. M. Barrie's latest literary contribution, "Sentimental Tommy."‖‖ The story is told in the writer's inimitable manner, and "Tommy" evokes strong sympathy in the breast of those who follow his fortunes through these pages.

* "Monte Christo." Alexander Dumas. (London: Walter Scott.)
† "Cassell's Family Magazine" for 1896. (London: Cassell and Co.)
‡ "Every Inch a Sailor." Gordon Stables. (London: Nelson and Sons.)
§ "To Central Africa on an Iceberg." Charles Squire and Frank Maclean. (London: Jarrold and Sons.)
‖ "Three Girls in a Flat." Ethel F. Heddle. (London: Gardner, Darton, and Co.)
¶ "A Humble Enterprise." Ada Cambridge. (London: Ward, Lock, and Bowden.)
** "The Little Larrikin." Ethel Turner. (London: Ward, Lock, and Bowden.)
†† "Iras: A Mystery." Theo. Douglas. (London: William Blackwood and Sons.)
‡‡ "Because of the Child." Curtis Yorke. (London: Jarrold and Sons.)
§§ "Some Unconventional People." Mrs. Gladwyn Jebb. (London: William Blackwood and Sons.)
‖‖ "Sentimental Tommy." J. M. Barrie. (London: Cassell and Co.)

### Christmas with the Women Students.

THE women students at the Royal Free Hospital, Gray's Inn Road, began their annual entertainments last week, and owing to the small size of the room in which the stage was erected they had to divide their guests and receive them on three successive nights, the programme being repeated. The performances were so excellent that it was unfortunate that a larger room could not be obtained. Old patients were known to be clamorous to see once more the clever entertainments of their friends, the lady students, but this could not be managed as there was barely room for present patients, staff, nurses, students, and their friends. The scenes from "The Rivals," which occupied the first part of the programme, were admirably given, both dresses and "make up" being excellent. It was hard to realise that that irascible old Sir Anthony Absolute was being played in such spirited fashion by Miss Anderson. Miss Williams made a most gallant young officer, and, in the character of Captain Absolute, was attractive in more eyes than Lydia's. Mrs. Malaprop was represented with much spirit by Miss Lewin, and Miss G. Stewart played the sentimental Lydia's part very sympathetically. Miss Fitter gave great pleasure by her singing in the second part, and "The Toyshop," arranged by Miss Williams and Miss Flint, was mirth-provoking from start to finish. Jack-in-the-Box, who was censured by the Fairy for his habit of frightening small children, and an old Dutch doll impersonated by Miss Patch, were alike clever. In fact, few real toy-shops could show such a charming array of pretty faces and dainty dresses as the women-students displayed. The boy-dolls of fierce military aspect were quite as well depicted, and their fragmentary conversations, after the fairy's wand had conferred temporary life on them, teemed with local allusions. A row of Chinese "Living pictures" were so clever that the audience wanted to see more of them than time permitted. Two topical songs proved that the Royal Free Hospital possesses at least one poetess. A song about "Two Little Maids" was well sung by Miss Roberts, who wore a becoming frilled sun-bonnet and represented the long haired doll with deep blue eyes dear to the heart of childhood. With this varied entertainment the Christmas treats have only begun, it seems, for carols have been sung in the wards on two evenings this week, and Christmas Day brings Christmas fare. The ward sisters give what a patient called "Fancy Teas" in their own domains. Trees and gifts and kindly greetings still pervade the hospital as we go to press, and we may fairly congratulate the women-students on their share in making Christmas, 1896, a happy one to many. Part of the programme of the entertainment at the Royal Free Hospital was repeated at the New Hospital for Women on 18th inst. by the students.

### Pirated Advertisement.

THE *Nursing Record* tried last week to be very funny in regard to a letter which appeared in our columns on December 12th complaining of that paper filling up its advertising columns by copying our old advertisements and thus misleading such persons as might happen to read the *Nursing Record* by tempting them to apply for posts after they had been filled up. Far be it from us to deny in any way that the *Nursing Record* is a funny paper, but if it wishes to be taken seriously as a journal for nurses, Mrs. Bedford Fenwick, who is stated to be its editor, should display something very different from mere fun and skittishness when her paper is accused of misleading her subscribers and giving annoyance both to matrons and to nurses by puffing out its columns with pirated advertisements. Either a denial, an explanation, or an apology might have been expected after so serious a complaint.

### For Reading to the Sick.

CHRISTMAS DAY.

"GLORY to God in the highest, and on earth peace, good will toward men."—*St. Luke ii.* 14.

**Verses.**

And so the Word had breath, and wrought
With human hand the Creed of creeds;
In loveliness of perfect deeds,
More strong than all poetic thought;
Which he may read who binds the sheaf,
Or builds the house, or digs the grave;
And those wild eyes that watch the wave
In roarings round the coral reef.
—*In Memoriam.*

Thou cam'st from Heaven to earth, that we
Might go from earth to Heaven with Thee;
And though Thou found'st no welcome here,
Thou did'st provide us mansions there.
—*H. Vaughan.*

Blest day which age reminds us, year by year,
What 'tis to be a man; to curb and spurn
The tyrant in us; that ignobler self
Which was no good save ease, no ill save pain,
No purpose, save its share in that wild war
In which through countless ages living things
Complete an internecine greed!
While ever out of the eternal Heavens
Looks down the great, magnanimous God,
Who, Maker of all worlds, did sacrifice—
All to Himself? Nay, but Himself to one;
Who taught mankind on that first Christmas Day
What 'twas to be a man; to give, to take;
To serve, not rule; to nourish, not devour;
To help, not crush; if need, to die, not live!
—*Kingsley.*

**Reading.**

"Wist ye not that I must be about My Father's business?"—*Luke ii.* 49.

"My meat and My drink are to do the will of Him that sent Me, and to finish His work." That *one* object brought Jesus from Heaven. That *one* object He pursued with unflinching, undeviating constancy, until He could say, "It is finished." However short man comes of *his* "chief end," "Glory to God in the Highest" was the motive, the rule, the exponent of every act of that wondrous life. With us, the magnet of the soul, even when truest, is ever subject to partial oscillations and depressions, trembling at times away from its great attractive point. His never knew one tremulous wavering from its all-glorious centre. With Him there were no ebbs and flows—no fits and starts. He could say, in the words of that prophetic psalm which speaks so pre-eminently of Himself, "I have set the Lord always before me!"

Reader, do you feel that, in some feeble measure, this lofty life-motto of the sinless Son of God is written on your home and heart, regulating your actions, chastening your joys, quickening your hopes, giving energy and direction to your whole being, subordinating all the affections of your nature to their high destiny. Seek to feel that your heavenly Father's is not only *a* business, but *the* business of life. "Whose I am, and Whom I serve"—let this be the superscription written on your thoughts and deeds, your employments and enjoyments, your sleeping and waking. Be not, as the fixed stars, cold and distant; but be ever bathing in the sunshine of conscious nearness to Him, Who is the sun and centre of all happiness and joy.—*From "The Words of Jesus."*

### Minor Appointments.

ROCHFORD WORKHOUSE INFIRMARY. — Nurse Adeline Kathleen Leonard has been appointed Nurse at this infirmary. Nurse Leonard was trained at the Belfast Union Infirmary, where she afterwards held the post of nurse for three years.

BIRMINGHAM AND MIDLAND EYE HOSPITAL.—Nurse Mabel Ferguson has been appointed Charge Nurse of the Children's Wing of this hospital. She was trained at the Bristol General Infirmary. Nurse E. Mathews has been placed in Charge of the Women's Wards, in succession to Nurse Jenny Gee, who has been appointed to the care of the male wards.

JAFFRAY BRANCH HOSPITAL.—Miss I. von Berg has been appointed Sister at this hospital. She was trained at the Seamen's Hospital, Greenwich, subsequently holding the posts of staff nurse at Huddersfield Infirmary, theatre and surgical sister at the Bristol Hospital for Sick Children and Women, and holiday sister at the Salop Infirmary. Miss von Berg has excellent testimonials.

CHESHIRE COUNTY ASYLUM, MACCLESFIELD.—Miss Howell has been appointed Head Nurse at this asylum. She received her training at the Bristol General Hospital, where she subsequently held the post of sister. Miss Howell then undertook the duties of night-superintendent at the Clayton Hospital, Wakeford, thence going as assistant head nurse to the Wilts County Asylum, Devizes.

### Notes and Queries.

*Training.*

(69) I am very anxious to enter one of the general hospitals for three years' training. Will you kindly advise me as to the steps to take to get into one? I have good health—*St. Heliers.*

You do not mention your age so we cannot tell you for which general hospitals you would be eligible. You will find a list of hospitals, London and provincial, in "How to Become a Nurse" (Scientific Press, 28 & 29, Southampton Street, Strand). Select the names of those which suit your requirements and for which you are eligible, and write to the matrons for application forms. Full particulars of the terms of admission and training at the principal London Hospitals have been given lately in the articles in the Nursing Supplement, "Nurses in 1896."

*District Nursing.*

(70) Is a year's general training sufficient for a district nurse?—*Question.*

A year's training in general nursing is required of women wishing to become "Queen's Nurses," to be followed by a six months' course of district training; for nurses in country places three months' approved training in midwifery is also required. You had better write to the Secretary, Queen Victoria's Jubilee Institute for Nurses, St. Katharine's Royal Hospital, Regent's Park, N.W.

*Stewardesses.*

(71) Please tell me where one should apply for particulars how to become stewardess on the P. and O. boats?—*A. S. H.*

Write to the offices of the P. and O. Company at 25, Cockspur Street, S.W.

*Homes for the Aged.*

(72) How can I find out if there is a home for the aged anywhere in the Midlands?—*Grannie.*

We cannot reply to anonymous queries. All communications requiring replies in this column must contain the full name and address of the sender (not for publication).

*Nursing Abroad.*

(73) I much wish to obtain a post as nurse or travelling companion abroad. Can you tell me how I could get this? I am nursing in Jersey for the winter, so have plenty of time before me. I have had three years' hospital training, and five years' experience in private nursing.—*K. J.*

We can only suggest that you should carefully watch the advertisements in our columns as well as in the daily papers, where such posts are now and again to be heard of. You might also advertise in THE HOSPITAL yourself. It has a large foreign circulation, and thus might lead to something. You should also acquaint your friends with your wishes, and especially the doctors for whom you have nursed. Before taking any post in a foreign country most careful inquiries should always be made. Many nurses have landed themselves in great difficulties through rashly venturing into engagements without due precautions.

*King's College Hospital.*

(74) Please tell me if nurse probationers at King's College Hospital have to produce a medical certificate of health from their own doctors?—*S. C.*

Address your question to Miss Monk, the matron of the hospital in question.

